# Supplementary material for: Plasma neurodegeneration biomarker concentrations associate with glymphatic and meningeal lymphatic measures in neurological disorders
Source: Nat Commun. 2023 Apr 12;14:2084. doi: 10.1038/s41467-023-37685-5 (PMC10097687; doi:10.1038/s41467-023-37685-5)
Supplement: Supplementary file 1 — Supplementary Information [file 41467_2023_37685_MOESM1_ESM.pdf]

Supplementary Material

Plasma neurodegeneration biomarker concentrations associate with glymphatic and meningeal lymphatic measures in neurological disorders

Per Kristian Eide,[1,2,*] Aslan Lashkarivand,[1,2] Are Pripp,[3,4] Lars Magnus Valnes,[1] Markus Herberg Hovd,[5] Geir Ringstad,[6,7] Kaj Blennow,[8,9] Henrik Zetterberg[8,9,10,11,12,13]

[1]Dept. of Neurosurgery, Oslo University Hospital-Rikshospitalet,
[2]Institute of Clinical Medicine, Faculty of Medicine, University of Oslo, Oslo, Norway
[3]Oslo Centre of Biostatistics and Epidemiology, Research Support Services, Oslo University Hospital, Oslo, Norway.
[4]Faculty of Health Sciences, Oslo Metropolitan University, Oslo, Norway.
[5]Section for Pharmacology and Pharmaceutical Biosciences, Department of Pharmacy, University of Oslo, Oslo, Norway
[6]Dept. of Radiology, Oslo University Hospital - Rikshospitalet,
[7]Department of Geriatrics and Internal medicine, Sorlandet Hospital, Arendal, Norway.
[8]Department of Psychiatry and Neurochemistry, Institute of Neuroscience and Physiology, the Sahlgrenska Academy at the University of Gothenburg, Mölndal, Sweden
[9]Clinical Neurochemistry Laboratory, Sahlgrenska University Hospital, Mölndal, Sweden
[10]Department of Neurodegenerative Disease, UCL Institute of Neurology, Queen Square, London, UK
[11]UK Dementia Research Institute at UCL, London, UK
[12]Hong Kong Center for Neurodegenerative Diseases, Clear Water Bay, Hong Kong, China
[13]UW Department of Medicine, School of Medicine and Public Health, Madison, WI, USA

***Correspondence:**
Professor Per Kristian Eide, MD PhD
Department of Neurosurgery
Oslo University Hospital - Rikshospitalet
Pb 4950 Nydalen, N-0424 Oslo, Norway
p.k.eide@medisin.uio.no/peide@ous-hf.no

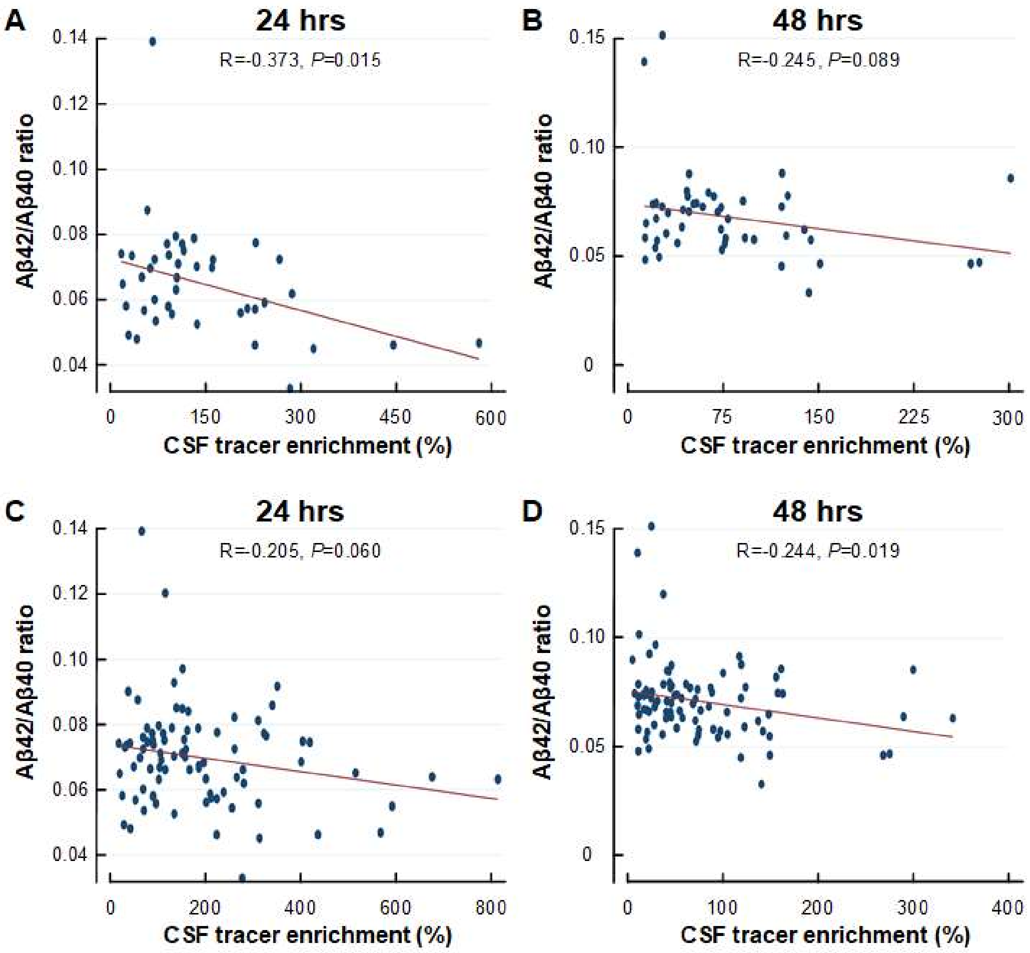

Supplementary Figure 1. Associations between plasma Aβ42/Aβ40 ratio and enrichment of CSF tracer within CSF spaces at 24 and 48 hours. In the iNPH cohort (**A**-**B**), plots of associations between plasma Aβ42/Aβ40 ratio and tracer enrichment in subarachnoid CSF spaces at (**A**) 24 hours and (**B**) 48 hours. In the total cohort (**C**-**D**), plots of associations between plasma Aβ42/Aβ40 ratio and tracer enrichment in subarachnoid CSF spaces at (**C**) 24 hours and (**D**) 48 hours. For each scatter plot, the fit line is shown, including Pearson correlations and significance levels. Higher tracer levels at 24 hours compared to Pre (*i.e.* higher % change normalized T1 signal) is reflecting reduced clearance of tracer (interpreted as impaired glymphatic function), which was associated with lower Aβ42/Aβ40 ratio, presumably due to reduced egress of Aβ42 to blood. Each scatter plot shows the linear fit line, including Pearson correlations and significance levels. Source data are provided as Source Data file.

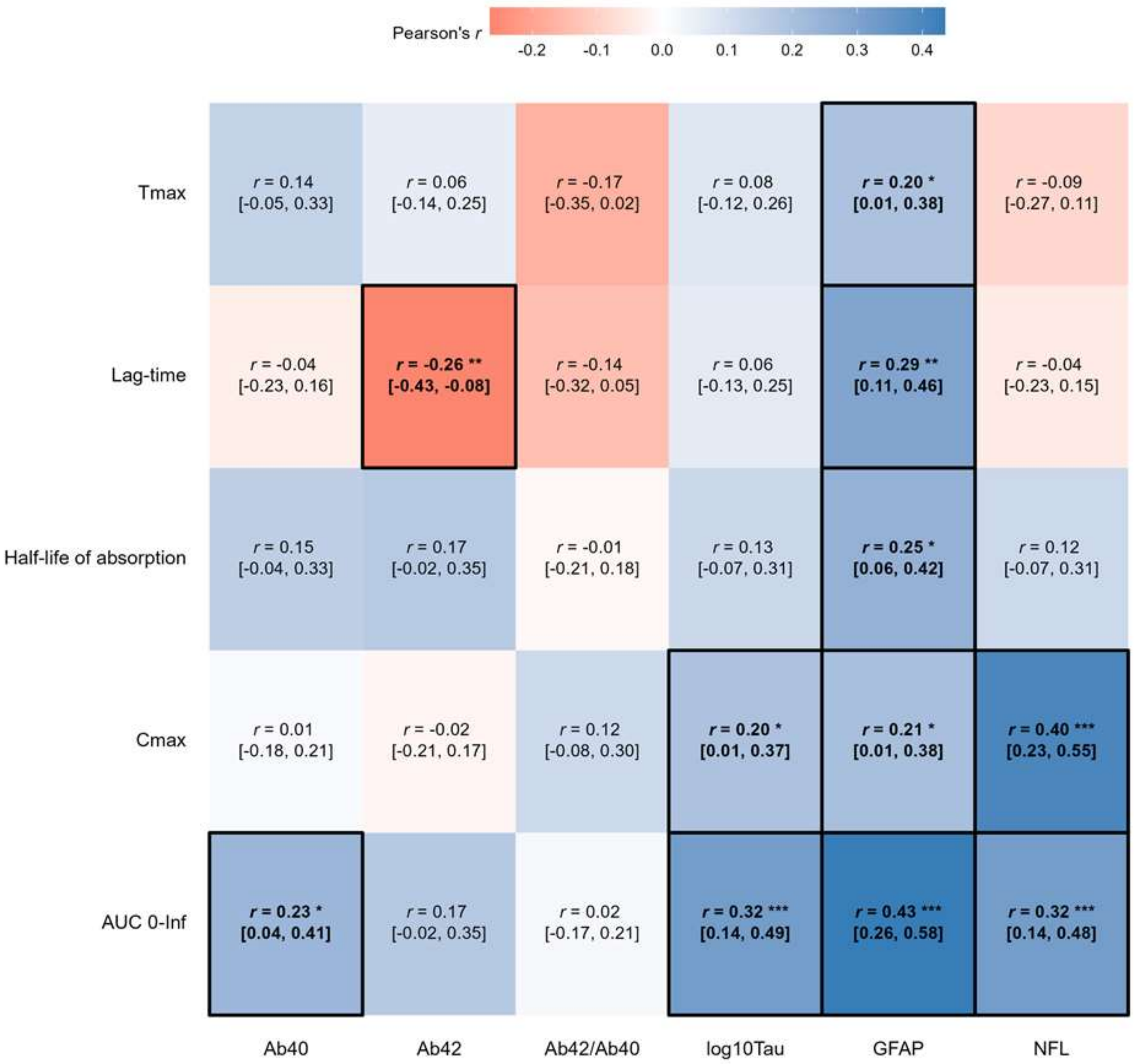

Supplementary Figure 2. Associations between CSF-to-blood clearance parameters and plasma biomarker concentrations for the total cohort of patients. The associations between CSF-to-blood clearance pharmacokinetic variables and plasma biomarker concentrations are shown as Pearson's correlations (*r*) [95% confidence interval] for the total cohort of patients included in the study. Significance levels of Pearson correlation coefficients: *P<0.05, **P<0.01, ***P<0.001 (values in bold are statistically significant to P < 0.05). Source data are provided as Source Data file.

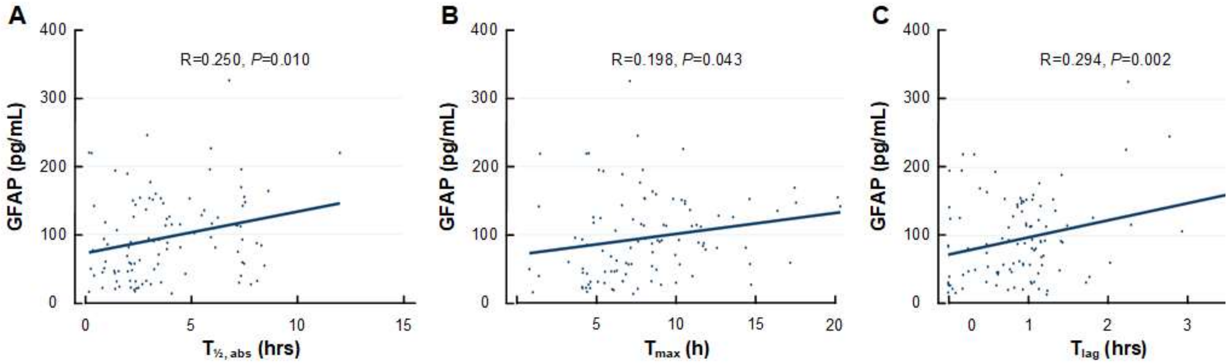

Supplementary Figure 3. Associations between plasma GFAP concentration and CSF-to-blood clearance variables derived from our pharmacokinetic model. There was a significant positive correlation between average plasma concentration of GFAP and (**A**) absorption half-life ($T_{1/2, abs}$), (**B**) time to maximum concentration (T_{max}), and (**C**) lag-time (T_{lag}). Hence, various measures of impaired CSF-to-blood clearance were associated with higher plasma GFAP concentration. Each scatter plots shows the fit line and the Pearson correlation coefficient with significance levels. Source data are provided as Source Data file.

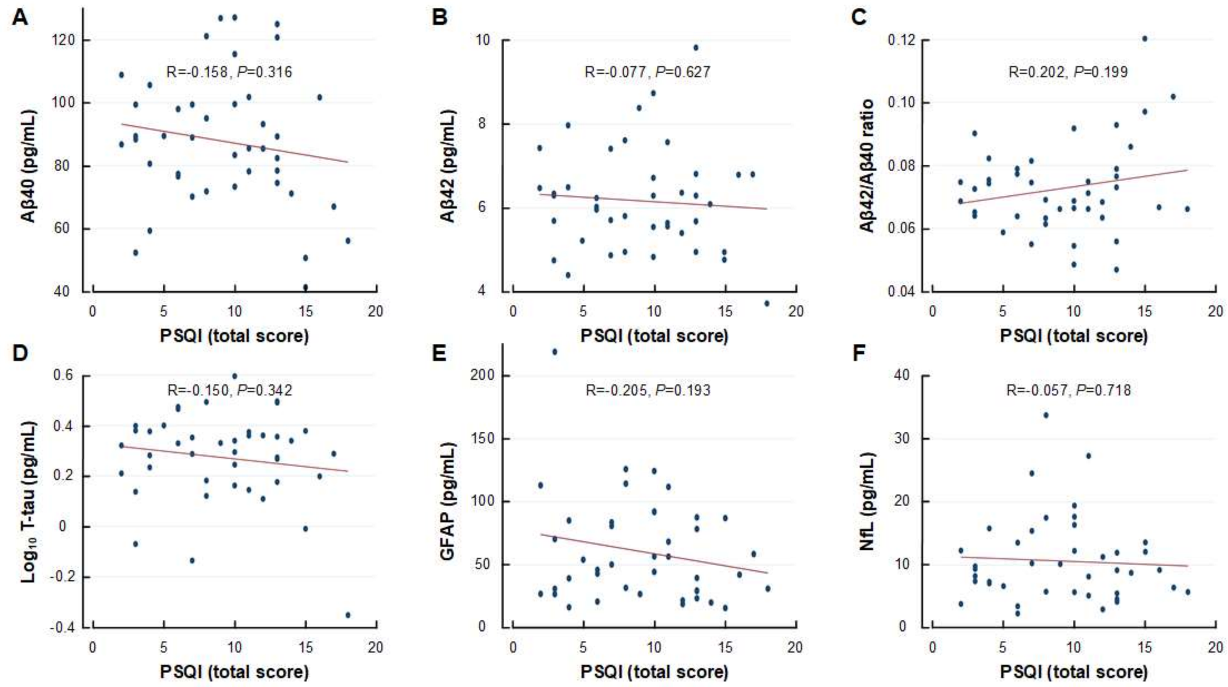

Supplementary Figure 4. Associations between subjective sleep quality, assessed by the total score of the Pittsburg sleep quality index (PSQI), and plasma biomarker concentrations. There were no significant correlations between subjective sleep quality (measured as total score of PSQI) and plasma concentrations of (A) Aβ40, (B) Aβ42, (C) Aβ42/Aβ40 ratio, (D) T-tau, (E) GFAP and (F) NfL. Each scatter plots shows the fit line and the Pearson correlation coefficient with significance levels. Source data are provided as Source Data file.

Supplementary Table 1. Differences in plasma metabolites between good (PSQI score ≤5) and poor sleepers (PSQI score >6)

Sleep group	Ab40	Ab42	Ab42/Ab40 ratio	Log10Tau	GFAP	NFL
**Good sleepers (n=10)**	85.97±18.25	6.11±1.12	0.073±0.009	0.27±0.15	68.28±61.13	8.78±3.32
**Poor sleepers (n=32)**	88.30±21.98	6.19±1.28	0.073±0.016	0.27±0.19	57.98±33.50	11.20±7.43

Data presented as mean±SD. No statistically significant differences between groups were seen (two sample t-test).
