# Supplementary material for: Plasma neurodegeneration biomarker concentrations associate with glymphatic and meningeal lymphatic measures in neurological disorders
Source: Nat Commun. 2023 Apr 12;14:2084. doi: 10.1038/s41467-023-37685-5 (PMC10097687; doi:10.1038/s41467-023-37685-5)
Supplement: Supplementary file 3 — Reporting Summary [file 41467_2023_37685_MOESM3_ESM.pdf]

nature portfolio

Corresponding author(s): Eide

Last updated by author(s): Mar 9, 2023

Reporting Summary

Nature Portfolio wishes to improve the reproducibility of the work that we publish. This form provides structure for consistency and transparency in reporting. For further information on Nature Portfolio policies, see our Editorial Policies and the Editorial Policy Checklist.

Statistics

For all statistical analyses, confirm that the following items are present in the figure legend, table legend, main text, or Methods section.

n/a	Confirmed	
☐	☒	The exact sample size (n) for each experimental group/condition, given as a discrete number and unit of measurement
☐	☒	A statement on whether measurements were taken from distinct samples or whether the same sample was measured repeatedly
☐	☒	The statistical test(s) used AND whether they are one- or two-sided<br>*Only common tests should be described solely by name; describe more complex techniques in the Methods section.*
☐	☒	A description of all covariates tested
☐	☒	A description of any assumptions or corrections, such as tests of normality and adjustment for multiple comparisons
☐	☒	A full description of the statistical parameters including central tendency (e.g. means) or other basic estimates (e.g. regression coefficient) AND variation (e.g. standard deviation) or associated estimates of uncertainty (e.g. confidence intervals)
☐	☒	For null hypothesis testing, the test statistic (e.g. F, t, r) with confidence intervals, effect sizes, degrees of freedom and P value noted<br>*Give P values as exact values whenever suitable.*
☒	☐	For Bayesian analysis, information on the choice of priors and Markov chain Monte Carlo settings
☐	☒	For hierarchical and complex designs, identification of the appropriate level for tests and full reporting of outcomes
☐	☒	Estimates of effect sizes (e.g. Cohen's d, Pearson's r), indicating how they were calculated

Our web collection on statistics for biologists contains articles on many of the points above.

Software and code

Policy information about availability of computer code

Data collection	FreeSurfer software (version 6.0) (http://surfer.nmr.mgh.harvard.edu/)
Data analysis	Strata/SE 17.0 (StrataCorp, College Station, TX)

For manuscripts utilizing custom algorithms or software that are central to the research but not yet described in published literature, software must be made available to editors and reviewers. We strongly encourage code deposition in a community repository (e.g. GitHub). See the Nature Portfolio guidelines for submitting code & software for further information.

Data

Policy information about availability of data

All manuscripts must include a data availability statement. This statement should provide the following information, where applicable:

- Accession codes, unique identifiers, or web links for publicly available datasets
- A description of any restrictions on data availability
- For clinical datasets or third party data, please ensure that the statement adheres to our policy

Source data file is accompanying the manuscript.

Human research participants

Policy information about studies involving human research participants and Sex and Gender in Research.

Reporting on sex and gender	The results refer to sex (female, n=56, male, n=50), not gender.
Population characteristics	The study was prospective and observational. Randomization was not relevant. Results are categorized according to diagnosis, which represents the most important covariate.
Recruitment	Consecutive patients referred to the Department of neurosurgery, Oslo university hospital - Rikshospitalet, Oslo, Norway, for various CSF disorders were included. The indication for intrathecal contrast enhanced MRI was made on clinical reasons. The inclusion criteria were clinically suspected cerebrospinal fluid disturbance. Exclusion criteria were evidence of renal dysfunction, allergy towards contrast agents, severe allergy in general, pregnant or breast feeding women, age <18 years /80 years
Ethics oversight	The research study was approved by The Institutional Review Board (2015/1868), Regional Ethics Committee (2015/96) and the National Medicines Agency (15/04932-7), and registered in Oslo University Hospital Research Registry (ePhorte 2015/1868).

Note that full information on the approval of the study protocol must also be provided in the manuscript.

Field-specific reporting

Please select the one below that is the best fit for your research. If you are not sure, read the appropriate sections before making your selection.

[x] Life sciences [ ] Behavioural & social sciences [ ] Ecological, evolutionary & environmental sciences

For a reference copy of the document with all sections, see nature.com/documents/nr-reporting-summary-flat.pdf

Life sciences study design

All studies must disclose on these points even when the disclosure is negative.

Sample size	Diagnosis is the most important covariate. N=8 was the lowest number of individuals in each diagnostic category, which we consider sufficient to address the present questions.
Data exclusions	All patients with blood samples and MRI were included, none were excluded.
Replication	Results are based on repeated blood samples and repeated MRI acquisitions. From 106 individuals 970 plasma samples , providing for analysis of the different neurodegeneration biomarkers.
Randomization	Randomization of patients was not relevant as this study did not compare different interventions.
Blinding	The personnel analyzing plasma biomarker concentrations and cerebral tracer enrichment were blinded to information about the study participants and all analyses were done separately without information about results from the various parts of the study.

Reporting for specific materials, systems and methods

We require information from authors about some types of materials, experimental systems and methods used in many studies. Here, indicate whether each material, system or method listed is relevant to your study. If you are not sure if a list item applies to your research, read the appropriate section before selecting a response.

Materials & experimental systems

n/a	Involved in the study	
[x]	[ ]	Antibodies
[x]	[ ]	Eukaryotic cell lines
[x]	[ ]	Palaeontology and archaeology
[x]	[ ]	Animals and other organisms
[x]	[ ]	Clinical data
[x]	[ ]	Dual use research of concern

Methods

n/a	Involved in the study	
[x]	[ ]	ChIP-seq
[x]	[ ]	Flow cytometry
[ ]	[x]	MRI-based neuroimaging

Magnetic resonance imaging

Experimental design

Design type	Prospective and observational
Design specifications	NA
Behavioral performance measures	NA

Acquisition

Imaging type(s)	MRI T1
Field strength	3T
Sequence & imaging parameters	Equal imaging protocol settings were applied at all time points to acquire sagittal 3D T1-weighted volume scans, with the following imaging parameters: repetition time = “shortest” (typically 5.1 ms), echo time = “shortest” (typically 2.3 ms), Flip angle = 8 degrees, field of view = 256 x 256 cm and matrix = 256 x 256 pixels (reconstructed 512 x 512). Hundred and eighty-four over-contiguous (overlapping) slices with 1 mm thickness were sampled, which were automatically reconstructed to 368 slices with 0.5 mm thickness. The duration of each image acquisition was 6 minutes and 29 seconds.
Area of acquisition	Whole brain scan

Diffusion MRI ☐ Used ☒ Not used

Preprocessing

Preprocessing software	FreeSurfer software (version 6.0) (http://surfer.nmr.mgh.harvard.edu/)
Normalization	Changes in the gray-scale between MRI scans were adjusted by dividing the T1 signal unit for each time point by the T1 signal unit of a reference region of interest (ROI) for the respective time point. The reference ROI was placed within the posterior part of the orbit, as previously described. The ratio is the normalized T1 signal units, which corrects for baseline changes of image gray scale due to automatic image scaling. Tracer enrichment was semi-quantified as percentage change in normalized T1 signal at different tome points, relative to pre-contrast injection.
Normalization template	Changes in the gray-scale between MRI scans were adjusted by dividing the T1 signal unit for each time point by the T1 signal unit of a reference region of interest (ROI) for the respective time point. The reference ROI was placed within the posterior part of the orbit, as previously described.
Noise and artifact removal	NA
Volume censoring	NA

Statistical modeling & inference

Model type and settings	Daytime variation in biomarker concentrations was analyzed using a non-linear model; a fractional polynomial linear regression with maximum one degree of the fractional polynomial and robust standard error for repeated measurements of the same subject. A general linear model or a two-sample t test assessed the mean difference between groups. Due to skewed T-tau values, we used the logarithm of T- tau values (log10T-tau).
Effect(s) tested	A general linear model or a two-sample t test assessed the mean difference between groups.

Specify type of analysis: ☐ Whole brain ☐ ROI-based ☒ Both

Anatomical location(s): Cerebral cortex, subcortical white matter, and cerebrospinal fluid.

Statistic type for inference (See Eklund et al. 2016)	NA
Correction	NA

Models & analysis

n/a	Involved in the study
☒	☐ Functional and/or effective connectivity
☐	☒ Graph analysis
☐	☒ Multivariate modeling or predictive analysis

Graph analysis	The plots were presented with the linear prediction (estimated mean from the regression model) and 95 %

	confidence interval.
Multivariate modeling and predictive analysis	Fractional polynomial regression analysis with robust standard error for repeated measurements, and regression analysis of average plasma biomarker concentrations.
